# An untargeted fecal and urine metabolomics analysis of the interplay between the gut microbiome, diet and human metabolism in Indian and Chinese adults

**DOI:** 10.1038/s41598-019-45640-y

**Published:** 2019-06-24

**Authors:** Abhishek Jain, Xin Hui Li, Wei Ning Chen

**Affiliations:** 1 Interdisciplinary Graduate School, Nanyang Technological University, 50 Nanyang Avenue, Singapore, 639798 Singapore; 2 Advanced Environmental Biotechnology Centre, Nanyang Environment & Water Research Institute, Nanyang Technological University, 1 CleanTech Loop, Singapore, 637141 Singapore; 3 School of Chemical and Biomedical Engineering, Nanyang Technological University, 62 Nanyang Drive, Singapore, 637459 Singapore; 4 Zhong Feng International, Hengyang City, China

**Keywords:** Metabolomics, Metabolomics

## Abstract

Gut microbiome plays a vital role in human health. Human fecal and urine metabolome could provide a functional readout of gut microbial metabolism as well as its interaction with host and diet. However, this relationship still needs to be fully characterized. We established an untargeted GC-MS metabolomics method which enabled the detection of 122 and 86 metabolites including amino acids, phenolics, indoles, carbohydrates, sugars and metabolites of microbial origin from fecal and urine samples respectively. 41 compounds were confirmed using external standards. Next, we compared the fecal and urine metabolome of 16 healthy Indian and Chinese adults, ages 22–35 years, using a combined GC-MS and LC-MS approach. We showed dietary habit or ethnicity wise grouping of urine and fecal metabolite profiles of Indian and Chinese adults. Our analysis revealed 53 differentiating metabolites including higher abundance of amino acids and phenolics in Chinese and higher abundance of fatty acids, glycocholic acid, metabolites related to tryptophan metabolism in Indian adults. Correlation analysis showed a strong association of metabolites with gut bacterial profiles of the same subjects in the genus and species level. Thus, our results suggest that gut bacterial compositional changes could be eventually monitored and probed using a metabolomics approach.

## Introduction

Metabolites mirror the health status of an individual by acquiring extensive insights into the functioning of a biological system. Metabolomics is a powerful technique that simultaneously detects hundreds of small molecules present in a given biological system such as fecal, urine or saliva samples^1^. Fecal metabolites are the final product of both cellular and microbial metabolism undergoing inside the human intestinal tract. Some of the metabolites from the gut are absorbed into the circulation and eventually chemically modified (that is, co-metabolized) by the host, then finally excreted with the urine^2^. The development of culture-free techniques, for example high-throughput DNA sequencing, suggests the presence of various microorganisms in human gastrointestinal tract which affects nutrient absorption, energy regulation, detoxification, or transformation of xenobiotics and the health of an individual^3–5^. It is known that gut microbiota composition is largely affected by diet, consequently metabolites produced by gut microbiota also determined by diet^6,7^. The complex gut microbial community utilizes both diet and host derived energy sources for growth, predominantly through fermentative metabolism^8^. Analysis of fecal and urine metabolic compositions has received a lot of attention, as it does not just reflect the status of the gut microbiome yet additionally bridge the connections between symbiotic microbes and the host’s health. Several previous studies have proposed the usefulness of fecal and urine metabolites in disease diagnosis, e.g. higher concentrations of amino acids, saturated fatty acids, and ursodeoxycholic acid was reported in fecal samples of colorectal cancer patients^9^. In another study higher levels of choline, trimethylamine N-oxide (TMAO) and betaine produced from dietary phosphatidylcholine in the gut were found to be associated with cardiovascular disease risk and atherosclerosis^10^. Moreover, some metabolites such as short chain fatty acids, phenolics and vitamins decrease the risk of the gastrointestinal disorder^11^, cancer^12^, diabetes^13^ and cardiovascular diseases^14^. Thus, examining the fecal and urine metabolomes serve as a vigorous strategy for understanding the interactions between diet, human metabolism, and the gut microbiota composition in health and disease.

In this regard, there is a growing need for developing a high-throughput and large-scale sample analysis method which is pivotal to the results of metabolomics in such a field. Several mass spectrometry-based techniques (MS) and nuclear magnetic resonances spectroscopy (NMR) have been employed to analyze metabolites levels in biological samples but GC-MS is the most robust method due to higher sensitivity, resolution, reproducibility and better reliability as compared to LC-MS and NMR. However, the choice of extraction solvents and derivatization method largely affect the simultaneous detection of the total number and different classes of metabolites within a single GC-MS analysis, thus it makes the sample preparation a tedious process^15^. In the last few years, the use of LC-MS in nutritional metabolomics has also been increasing. LC-MS is more suitable for labile compounds and in addition to those that are difficult to derivatize^16^. An untargeted global investigation of urine or fecal samples is useful to identify metabolite biomarkers of diet or disease.

The two most populated Asian countries, India and China, have a unique diet profile. In our previous study, we determined the gut microbiota composition of healthy Indian and Chinese adults. In this study, an untargeted GC-MS metabolomics method was established for fecal and urine samples. Untargeted GC-MS and LC-MS metabolite profiling were performed on 16 fecal and urine samples obtained from 11 Indian and 5 Chinese adults. Dietary habits or ethnicity wise grouping of subjects were observed based on their metabolite profiles. Next, we performed a correlation analysis between metabolites and gut bacteria.

## Results and Discussion

We established an untargeted GC-MS metabolomics method for fecal samples using two solvent extraction steps, first acetonitrile: methanol followed by methanol: water, with trimethylsilylation derivatization. Although methanol alone has been proved to be a suitable solvent for metabolite extraction from human biofluids but most of these methods did not consider the importance of protein precipitation step in the fecal GC-MS analysis^17^. Precipitation of protein form fecal samples can be achieved by adding salts or acids but the addition of water miscible solvents prior to GC-MS could be a better approach. It decreases the electrolyte which improves MS sensitivity and avoids instrument capillary blockage^18^. The solubility of phospholipids in methanol is high, hence, in the event that methanol is utilized as an extraction solvent, lipids (including triacylglycerides and phospholipids) are extracted in sizable quantity which are involatile in GC-MS under trimethylsilylating conditions and would, consequently increase the carry-over background fatty acid signals detected in the chromatograms^19–21^. We chose acetonitrile as it is more effective in removing the phospholipids due to poor solubility of phospholipids in acetonitrile^22,23^. The second step with just methanol was likely to get a more complete extraction and as proteins already denatured and precipitated in the first step, it cannot get into solution. Moreover, the nonpolar nature of methanol as a solvent can help maximize the metabolome coverage. Also, methanol is an effective desalting agent.

To study the metabolic activity in the gut ecosystem of healthy humans and understand the relationship between diet, gut microbiome and fecal or urine metabolites, we applied our GC-MS method on the fecal samples of 16 healthy Asian adults. We extracted 122 metabolites including amino acids, phenolics, indoles, dicarboxylic acids and other metabolites of microbial origin (Table 1). The same method was applied to urine samples and it enabled the detection of 86 metabolites as listed in the Table 1. There were 60 metabolites commonly found in both fecal and urine samples which suggest that urine could also be used as a non-invasive tool to monitor the functional status of the gut microbiome. Two technical replicates were run for each sample and only the metabolites detected in both the cases were listed, which shows the reproducibility of the GC-MS method. Out of all the metabolites detected, the presence of 41 compounds was further confirmed using commercial external standards. The representative GC-MS chromatogram of the fecal extract is shown in Fig. S1.

**Table 1:** List of metabolites detected in fecal and urine samples of Indian and Chinese adults using GC-MS metabolomics.

RT	Fecal Metabolites	Urine metabolites		Origin
7.9	Lactic acid		confirmed	Microbial
8.26	Acetic acid			Microbial
8.58	2-propenoic acid			
9.07	L-alanine		confirmed	
10.22	Propanedioic Acid			
10.84	3-hydroxybutyric acid			
11.89	Cyclohexanecarboxylic acid			
12.67	L-valine		confirmed	
13.4	Benzoic acid		confirmed	Microbial
14.58	L-leucine		confirmed	
14.93	3-pyridinecarboxylic acid			
15.14	Benzeneacetic acid			Microbial
15.27	L-isoleucine		confirmed	
15.6	Glycine	Glycine	confirmed	
15.88	Succinic acid	Succinic acid	confirmed	Microbial
16.26	Methylsuccinic acid	Methylsuccinic acid		
16.47	n-valeric acid	n-valeric acid		Microbial
16.68	Pyrimidine	Pyrimidine		
16.89	2-butenedioic acid			
17.17	4-hydroxybenzaldehyde	4-hydroxybenzaldehyde	confirmed	
17.25	5-hydroxyhexanoic acid			
17.33	Pipecolic acid	Pipecolic acid		Microbial
17.5		2,3-Dihydroxybutanoic acid		
17.61	Serine	Serine	confirmed	
17.94	benzene			
18.47	L-threonine	L-threonine	confirmed	
18.75	Pentanedioic acid			Microbial
18.83	3- phenylpropionic acid		confirmed	
19.5	Beta-alanine	Beta-alanine		
19.6	Indole		confirmed	Microbial
19.9	3,4-dihydroxybutanoic acid	3,4-dihydroxybutanoic acid		
20.05	Propylene glycol			
20.35	L-homoserine	L-homoserine		Microbial
20.99	Pyruvic acid			
21.63	Malic Acid		confirmed	
21.75	4-pentenoic acid			
21.8	2-pyrrolidone-5-carboxylic acid			
21.85	Hexanedioic acid			
21.98	2-aminocaprylic acid			
22.08	pyroglutamic acid			
22.25		Butane		
22.31	L-methionine	L-methionine	confirmed	
22.36	L-proline		confirmed	
22.5	L-aspartic acid	L-aspartic acid	confirmed	
22.53		Pyrogallol		
22.76	4-methoxyphenylacetic acid	4-methoxyphenylacetic acid	confirmed	
22.78	Gamma-Aminobutyric acid		confirmed	
22.8	trans-Cinnamic acid		confirmed	Microbial
22.83		2-Furancarboxylic acid		
22.93		Creatinine		
23.35	L-cysteine		confirmed	
23.48		2,3,4-trihydroxybutyric acid		
23.51	Dodecanol			
24.07	L-threonic acid	L-threonic acid		
24.11	Linolenic acid	Linolenic acid		
24.72	3-hydroxybenzoic acid	3-hydroxybenzoic acid	confirmed	Microbial
24.9	Cyclohexylacetate			
25.14	L-ornithine			
25.18	4-hydroxybenzoic acid	4-hydroxybenzoic acid	confirmed	Microbial
25.26	Phenylalanine		confirmed	
25.34	L-glutamic acid		confirmed	
25.37		Mannonic acid		
25.45	Acetamide			
25.48		2,3-dimethyl-3-hydroxyglutaric acid		
25.57	3,5-dihydroxybenzoic acid	3,5-dihydroxybenzoic acid		
25.76	4-hydroxybenzeneacetic acid	4-hydroxybenzeneacetic acid	confirmed	Microbial
26.26		Tartaric Acid		
26.31	Arachidonic acid			
26.57	Phenol	Phenol		
26.7	3,4,5-trihydroxy pentanoic acid	3,4,5-trihydroxy pentanoic acid		
26.89	2-propenoic acid	2-propenoic acid		
26.96	D-arabinonic acid	D-arabinonic acid		
27.02	d-xylose	d-xylose		
27.45	5-hydroxyindole	5-hydroxyindole		
27.63		Arabinitol		
27.69	Threitol			
27.72	Glycyl-1-glutamic acid			
27.8	1,6-anhydro-.beta.-d-glucose	1,6-anhydro-.beta.-d-glucose		
27.83		L-Arabinose		
27.93	3-(3-hydroxyphenyl)propanoic acid	3-(3-hydroxyphenyl)propanoic acid		Microbial
28.2	3-hydroxyhex-2-enedioic acid			
28.23	1,4-butanediamine	1,4-butanediamine		Microbial
28.35		acetamide		
28.43	Tricarballylic acid			Microbial
28.53		d-(+)−Arabitol		
28.65	Adonitol	Adonitol	confirmed	
28.85	Hydrocinnamic acid	Hydrocinnamic acid	confirmed	Microbial
29	trans-Aconitic acid	trans-Aconitic acid		
29.25	Phenylacetic acid	Phenylacetic acid		Microbial
29.6	Phosphoric Acid	Phosphoric Acid		
29.81	Azelaic Acid	Azelaic Acid		
30.11	Ribonic Acid	Ribonic Acid		
30.64		L-sobopyronase		
30.88	Cadaverine			Microbial
30.93	D-Arabinose			Microbial
31.05		1,2,3-propanetricarboxylic acid		
31.06	3,4 -dihydroxyphenylacetic acid		confirmed	Microbial
31.13		(4-hydroxy-3-methoxyphenyl) ethylene glycol		
31.22	N-alfa-acetyl-L-lysine			
31.33	1H-Indole-3-ethanamine			
31.38		Pinitol		
31.45	3-(3-hydroxyphenyl)-3-hydroxypropionic acid			
31.55	Citric acid	Citric acid	confirmed	
31.62	Ethylmalonic acid	Ethylmalonic acid		
32.01	Hydrobenzoin			
32.14	Arabinitol	Arabinitol		
32.23		Gluconolactone		
32.3	3-hydroxy-3-phenylpropanoic acid	3-hydroxy-3-phenylpropanoic acid		
32.48	L-(-)-Sorbose	L-(-)-Sorbose		
32.64	Galactonic acid	Galactonic acid		
32.85	D-Fructose	D-Fructose	confirmed	
32.91	d-Galactose	d-Galactose	confirmed	
33.14	d-Glucose	d-Glucose	confirmed	
33.2	L-Lysine		confirmed	
33.35	Tyrosine		confirmed	
33.57	d-Mannose	d-Mannose		
33.64	3,4-dihydroxyhydrocinnamic acid	3,4-dihydroxyhydrocinnamic acid	confirmed	Microbial
33.88	3-(4-hydroxyphenyl)propionic acid	3-(4-hydroxyphenyl)propionic acid	confirmed	
33.92	1H-indole-3-Acetic Acid		confirmed	Microbial
33.99	D-mannitol	D-mannitol	confirmed	Microbial
34.15	Dulcitol	Dulcitol		
34.4	MyoInositol	MyoInositol		
34.53	Aniline			
34.93	Pantothenic acid	Pantothenic acid		
35.2	Dextrose		confirmed	
35.56		D-Gluconic acid		
35.72	Hexadecanoic acid	Hexadecanoic acid		
35.78	(3,4-dihydroxy phenyl)pentanoic acid			
36.15	Scyllo-Inositol	Scyllo-Inositol		
36.41	cis-5,8,11-Eicosatrienoic acid	cis-5,8,11-Eicosatrienoic acid		
36.57	3-Indolepropionic acid			Microbial
37.4	Inositol	Inositol		
37.88	Heptadecanoic acid	Heptadecanoic acid		
38.14	Sedoheptulose	Sedoheptulose		
38.63	D-Arabinopyranose	D-Arabinopyranose		
38.73	D-Glucitol	D-Glucitol		
38.95	5-hydroxyindolepropionic acid			
40.06	Octadecanoic acid	Octadecanoic acid		
46.96	Hexacosane	Hexacosane		
49.15	Sucrose	Sucrose	confirmed	
49.94		cellobiose		
50.51	maltose	maltose		

41 metabolites confirmed using analytical standards and metabolites of microbial origin are shown.

**Figure 1:**
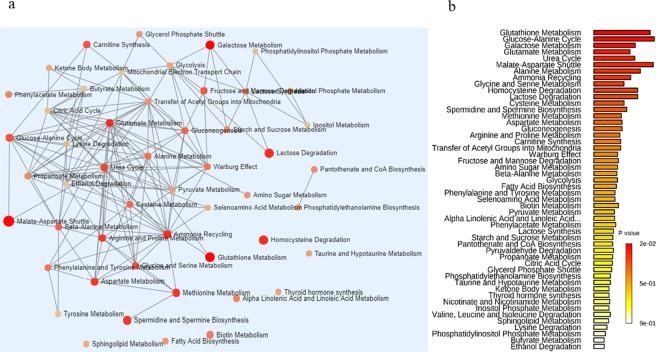
A network map and a bar graph with the functional role of metabolites detected using GC-MS metabolomics in fecal and urine samples of Indian and Chinese adults.

In order to further understand the usefulness of the metabolites detected with the GC-MS method, we performed pathway analysis to relate the metabolites with their corresponding pathways. A network map, a bar graph and a table with the probable functional role of metabolites are generated (Table S2, Fig. 1a,b). The functional analysis of urine and fecal metabolites of Indian and Chinese adults showed that most of the metabolites were involved in more than one pathway. For example, the metabolites of glutamate metabolism (Gamma-Aminobutyric acid, glycine, L-glutamic acid, L-alanine, L-aspartic acid, pyruvic acid, succinic acid, L-cysteine, phosphoric acid) represented most in the study. The metabolites related to arginine and proline metabolism were Glycine, L-glutamic acid, L-proline, L-aspartic acid, ornithine, succinic acid, phosphoric acid.

### Fecal and urine metabolomics revealed differences between Indian and Chinese adults

The Indian adults recruited for this study were consuming food which closely matches to the Mediterranean style diet. The main components of their diet were whole wheat or other whole grains, nuts, rice, lentils, legumes, green vegetables, fruits, dairy products, sweets, ghee, refined flour and fast food. On the other hand, Chinese adults consumed a diet including seafood, fish, chicken, pork, beef, a lot of variety of other meat, which was high in animal fat and protein. In addition, rice, noodles, beans, peanut oil, green vegetables, white flour, refined grains were a substantial part of Chinese diet (Table 3). In our previous study, we have reported the differences in gut microbiome composition between Indian and Chinese adults^24^. To assess whether the differences in gut microbiome composition and dietary habits between Indian and Chinese adults can alter the luminal environment, GC-MS and LC-MS metabolomics were performed on 16 fecal and urine samples, including 11 Indian and 5 Chinese. Overall 69 and 123 metabolites were extracted from LC-MS metabolomics of fecal and urine samples, respectively (Table S3). We used a combination of both GC-MS and LC-MS for more comprehensive metabolomics analysis^25^.

**Figure 2:**
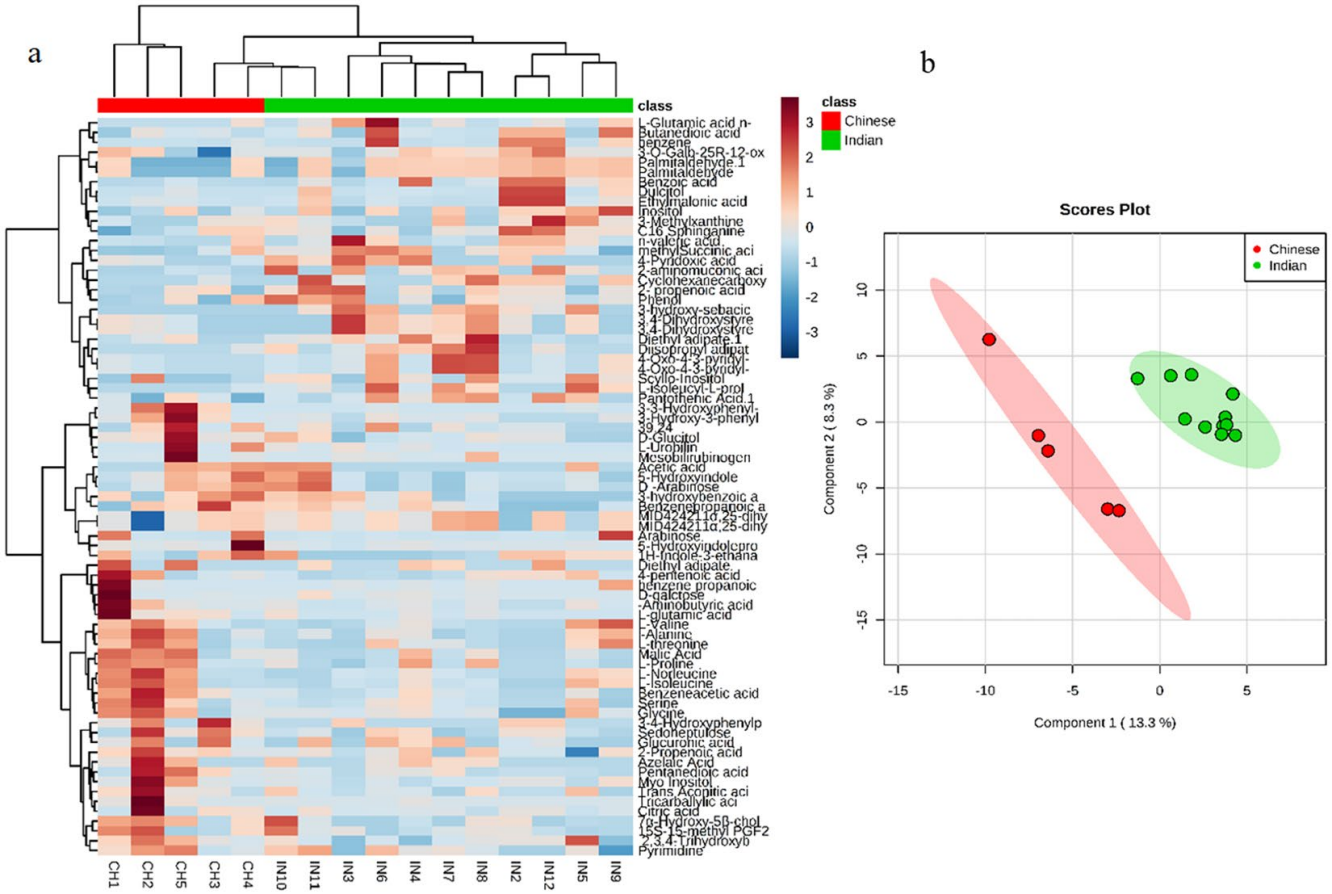
Subjects are clustered based on their dietary habits or ethnicity. (**a**) Heat map of the distribution of fecal metabolites among all individuals. (**b**) Partial least square discriminant analysis of fecal metabolites profiles of Indian and Chinese adults.

A simple average was used for the metabolites identified in both GC-MS and LC-Ms analysis. The multivariate statistical analysis was applied on data of fecal metabolites. Partial least square discriminant analysis plot and hierarchical clustering analysis heatmap showed dietary habit wise clustering of subjects. PLS-DA plots based on Leave- one- out cross-validation had R^2^ = 0.96 and Q^2^ = 0.683 which indicates total explained variance and cross validation predictive ability, respectively. The heatmap representing the distribution of fecal metabolites among all the individuals and PLS-DA plots are shown in Fig. 2a,b.

**Figure 3:**
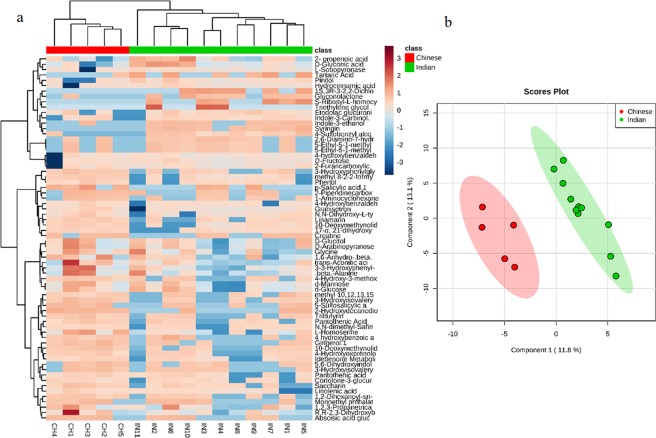
Urine metabolites profiles are influenced by dietary habits or ethnicity of subjects. (**a**) Heat map of the distribution of urine metabolites among all individuals. (**b**) Partial least square discriminant analysis of urine metabolites profiles of Indian and Chinese adults.

In order to further strengthen our results, the multivariate statistical analysis was applied on urine metabolites profiles also. PLS-DA plot with R^2^ = 0.80.81, Q^2^ = 0.631 and heat map of urine metabolites data also showed a clear distinction between Indian and Chinese based on their urine metabolite profiles. The heatmap representing the distribution of urine metabolites among all the individuals and PLS-DA plots are shown in Fig. 3a,b.

Overall dietary habits or ethnicity were found to play an important role in clustering of individuals based on urine and fecal metabolite profile. These results justify our previous findings where diet or ethnicity was found to be important in determining gut microbiome composition^24^.

### Metabolites that differentiate between Indian and Chinese adults could be associated with their diet

It has been reported that variable importance in the projections (VIP) values greater than 1 could be considered as the most relevant metabolites for explaining the differences^26^. Based on the criteria of VIP >1, 53 compounds distinguishing Indian and Chinese adults were identified. The metabolites with VIP values and fold change ratio between Indian and Chinese are presented in Table 2. In order to remove the gender based biases in our results, we also analysed the metabolites that differentiate between subjects based on gender (Table S1). It was found that only four metabolites (proline, homoserine, 3-hydroxy-3-phenylpropanoic acid, L-Urobilin) were commonly affected by both gender and dietary habits.

**Table 2:** Metabolites that differentiate between Indian and Chinese adults.

Metabolite	VIP	Fold (Chinese/Indian)
L-alanine	1.71	1.72
L-leucine	1.87	3
L-isoleucine	1.73	2.06
Glycine	1.62.	1.871
L-proline	1.67	2.2
Serine	1.75	2.62
L-glutamic acid	1.69	9.84
L-threonine	1.22	1.7
Gamma aminobutyric acid	1.71	2.65
L-homoserine	1.81	2.18
Benzeneacetic acid	2.34	3.96
3-(4-hydroxyphenyl) propionic acid	1.67	3.94
3-hydroxy-3-phenylpropanoic acid	1.93	4.66
3-(3-hydroxyphenyl)-3-hydroxypropionic acid	2.09	37.09
3-hydroxybenzoic acid	1.43	2.18
Malic acid	2.03	4.26
Citric acid	1.36	3.03
Sedoheptulose	1.76	2.56
5-hydroxyindole	1.3	1.76
2-hydroxydecanedioic acid	2.39	3.7
L-urobilin	1.59	7.92
3-hydroxyisovalerylcarnitine	1.82	6.98
Pentanedioic acid	1.97	3.72
Tricarballylic acid	1.46	29.72
3-hydroxyphenylglycine	1.69	3
Myoinositol	1.74	4.09
2-piperidinecarboxylic Acid	1.25	4.13
5,6-dihydroxyindole	0.98	3.26
4-sulfobenzyl alcohol	2.49	0.229
5-sulfosalicylic acid	1.26	4.12
L-sobopyronase	1.32	0.7
D-gluconic acid	1.97	0.54
Dulcitol	1.11	0.085
Tartaric Acid	1.30	0.008
L-isoleucyl-L-proline	1.54	0.082
Pantothenic acid	1.51	0.47
Palmitaldehyde	1.54	0.43
Diisopropyl adipate	1.41	0.166
Cyclohexanecarboxylic acid	1.541	0.106
S-ribosyl-L-homocysteine	1.86	*
Syringin	2.73	*
4-pyridoxic acid	1.22	0.38
Indole-3-ethanol	1.73	0.45
2-aminomuconic acid semialdehyde	1.65	0.11
3-hydroxy-sebacic acid	1.95	0.10
Glycocholic Acid	1.28	0.51
D-glucitol	1.55	2
Pinitol	2.07	0.21
Gluconolactone	1.78	0.08
Benzoic acid	1.49	0.39
3-phenylpropionic acid	1.32	3.89
Creatinine	1	1.31
Creatine	2.31	1.2

All the metabolites are presented with variable importance in projection (VIP) values and fold change ratio (Chinese/Indian). Metabolites affected by both diet and gender are mentioned in red.

*Detected only in Indian samples.

The levels of 7 amino acids were higher in Chinese adults, consistent with high protein consumption in subjects consuming Chinese diet. Our results are in agreement with the study carried out by Shankar *et al*. (2017) where US children consuming the western diet with high protein showed a higher level of amino acids as compared to Egyptian consuming Mediterranean diet^27^. Microbial degradation of dietary proteins results in the production of amino acids. However, the bioavailability of amino acids in the host is controlled by the gut microbiota composition. It has been reported previously that distribution of free amino acids in the gastrointestinal tract of germ free and conventionalized mice can be altered by the gut bacteria as the amino acids could be utilized by the bacteria in SCFA synthesis^28,29^. Pathway analysis on differentiating metabolites showed that the seven amino acid metabolites (glycine, L-serine, L-isoleucine, L-proline, L-glutamic acid, L-alanine, L-threonine) were found to be involved in aminoacyl-tRNA biosynthesis pathway. The etiology of diseases including cancer, neuronal pathologies, autoimmune disorders and abnormal metabolic conditions is found to be associated with aminoacyl-tRNA synthetases^30^. The metabolites L-glutamic acid, Gamma-Aminobutyric acid, L-alanine were associated with alanine, aspartate, and glutamate metabolism. Glycine, L-glutamic acid, L-alanine were related to glutathione metabolism and alanine metabolism. Metabolites of glutamate metabolism (Gamma-Aminobutyric acid, glycine, L-glutamic acid, L-alanine) were higher in Chinese. Another group of five metabolites (L-threonine, L-serine, glycine, creatine, L-homoserine) was associated with glycine, serine and threonine metabolism.

We have also noticed a marked increase in benzeneacetic acid in Chinese samples which has previously been associated to high protein diet^31,32^. Interestingly, most of the phenolics (3-(4-hydroxyphenyl) propionic acid, 3-hydroxy-3-phenylpropanoic acid, 3-(3-hydroxyphenyl)-3-hydroxypropionic acid, 3-hydroxybenzoic acid, 3-phenylpropionic acid) were higher in Chinese except benzoic acid which was more abundant in Indian subjects. The high amount of phenolics could be associated with high consumption of soy products, eggplants, mushrooms, blueberry, cranberry and leafy green vegetables such as broccoli, cabbage, cauliflower, spinach^33^. It is evidenced that phenolics are derived from the plant-based diet but these compounds can also be produced from microbial fermentation of protein-rich diet^31,32^. Phenolics displayed many important functions including inhibition of pathogens, prevention of various chronic diseases such as cancer, diabetes, and cardiovascular diseases, antioxidant^34,35^. Levels of central metabolism intermediates (malate, citrate, sedoheptulose, myoinositol, and D-Glucitol) were also higher in Chinese samples, possibly indicating incomplete fermentation of complex polysaccharides in the guts of these adults. Furthermore, higher abundance of creatine and creatinine in Chinese adults concurs with Stella *et al*. (2006), which showed consumption of meat is associated with an increase of these metabolites^36^. Creatinine is a breakdown product of creatine and high levels of creatinine could be an indicator of kidney diseases^37^.

The higher amount of fatty acids and conjugates (palmitaldehyde, 3-hydroxy-sebacic acid, diisopropyl adipate) were observed in Indian samples. The higher amount of fatty acids could be associated with higher secretion of glycocholic acid in Indians. It is a secondary bile acid produced by the bacterial actions in the colon. Bile acids act as a fatty acid emulsifier to facilitate the digestion of fats and oil. The levels of metabolites related to tryptophan metabolism (2-aminomuconic acid semialdehyde, Indole-3-ethanol) were also higher in Indians. Higher abundance of tryptophan related metabolites has been associated with the Mediterranean diet^38^. The bioactive compound, a phenylpropanoid, syringin was detected only in Indian subjects which could be linked with plant based diet. Syringin is known for its pharmacological properties including scavenging of free radicals, anti-diabetic effect, anti-allergic effect, anti-inflammatory potential^39^.

**Figure 4:**
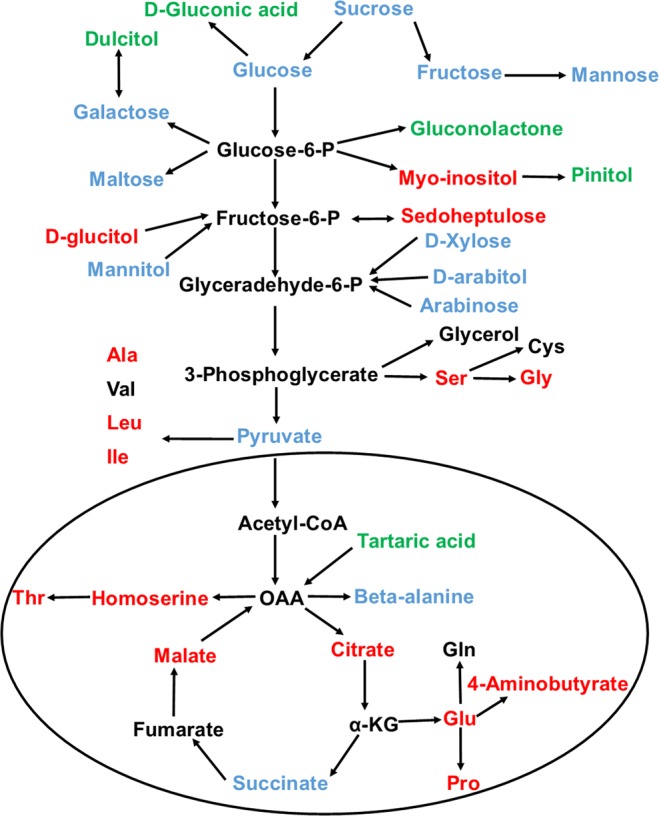
Metabolites that differentiates Indian and Chinese adults are mapped onto metabolic pathways. Red color represents higher abundance in Chinese, green color represents higher abundance in Indian, blue color shows the metabolites that do not differ between the two groups and black represents the undetected metabolites.

The alteration of metabolites in response to different dietary components has been studied but linking the metabolite changes to specific pathways still remains a challenge. The effect of diet on the human body and health or diseased status are directly correlated with the presence or absence of specific combinations of metabolites. Generally, it is the combination of metabolites rather than the individual compounds, which is of great biological relevance^40^. Therefore, the alteration in the metabolites involved in the central metabolism and in the linking metabolites to amino acid synthesis were respectively summarized on a simplified metabolic map (Fig. 4).

### Gut microbiome is correlated with fecal and urine metabolites

We investigated the interactive features between metabolites differentiating Indian and Chinese adults, metabolites of microbial origin and gut microbiome. The metabolites showed a comprehensive correlation with available 16S rRNA sequencing data on the gut bacterial profiles of the same subjects in the genus and species level^24^. The coefficient of correlation between gut bacterial profile and metabolites are given in Table S4.

Three genera, *Ruminococcus, Dorea*, and *Blautia* which are a member of one of the most abundant families, *Lachnospiraceae*, are positively correlated with L-alanine, L-leucine, L-isoleucine, glycine, serine, and L-proline. Consistent with our results, in a recent study of Swedish adults, all these genera were associated with increased levels of amino acids except a negative correlation was observed with serine^41^. Similarly, *Clostridium* which is known to be involved in the amino acid production^42^ was found to be associated with L-alanine, L-norleucine, L-isoleucine, glycine, serine, L-proline in our study. Genus *Turicibacter* and species *Bifidobacterium longum*, *Lactobacillus mucosae*, *Lactobacillus zeae* were positively correlated with 3,4-dihydroxyhydrocinnmaic acid. *Bifidobacterium* and *Lactobacillus* have been reported to be associated with hydroxycinnamic acid and polyphenol production^43–45^. Similarly, a positive correlation of *Eubacterium* with 3-hydroxybenzoic acid and 4-hydroxybenzoic acid concurs with the previous studies^46^. *Bacteroides ovatus* is correlated with n-valeric acid. *Collinsella aerofaciens* showed a positive correlation with sedoheptulose, citric acid, tricarballylic acid. *E. coli* was positively correlated with pipecolic acid. Genus *Faecalibacterium* showed a negative correlation with L-leucine and serine and species *Faecalibacterium prausnitzii* showed a negative correlation with serine, L-leucine, and malic acid. *Lactobacillus ruminis* showed a negative correlation with glycine. Genera *Dialister, Catenibacterium, Turicibacter* showed a positive correlation with tartaric acid and species *Ruminococcus bromii, Parabacteroides distasonis, Bacteroides caccae* were negatively correlated with tartaric acid. Genera *Akkermansia*, species *Coprococcus catus* were positively correlated with creatine and genera *Mitsuokella, Weissella, Lactobacillus*, species *Mitsuokella multacida*, *Lactobacillus ruminis* were negatively correlated with creatine. Genera *Faecalibacterium*, *Succinivibrio*, *Macrococcus* are negatively correlated with creatinine. Genera *Bilophila*, *Enterococcus*, *Dorea*, *Clostridium*, *Phascolarctobacter* and species *Faecalibacterium prausnitzii, Photobacterium angustum* are positively correlated with creatinine. Genera *Escherichia, Paraprevotella, Akkermansia* have a positive correlation with glycocholic acid. Genus *Carnobacterium* and species *Ruminococcus flavefaciens, Butyricicoccus pullicaecorum, Mitsuokella multacida*, *Bacteroides fragilis* have a negative correlation with glycocholic acid. Genera *Enterococcus*, *Dermacoccus*, *Kocuria*, *Roseburia* and species *Eggerthella lenta*, *Bacteroides fragilis* showed a negative correlation with 3-phenylpropionic acid. Genus *Oscillospira* is positively correlated with citric acid, concurs with Santoru *et al*. (2017). Genera *Acinetobacter, Microbacterium*, *Dysgonomonas*, *Bulleidia*, *Oscillospira* and species *Bacteroides caccae*, *Kocuria rhizophila*, *Pseudomonas fragi*, *Ruminococcus bromii* were positively correlated with 4-pyridoxic acid.

## Conclusions

In summary, we have established a trimethylsilylation based GC-MS metabolomics method which enabled the detection of various important fecal and urine metabolites such as amino acids, phenolics, indoles, sugars, in health and nutritional studies. There are very few studies reported the direct comparison of fecal and urine metabolites. Most of the studies on the  gut microbiome are based on fecal metabolome but the presence of significant number of common metabolites in our urine sample suggests that urine could also be used as an important non-invasive tool to monitor the functional status of gut microbiome. Dietary habit or ethnicity were found to play an important role in determining gut microbiome and metabolites composition in our study. The present study was based on long-term dietary habits with a very limited number of sample size. Other hidden factors such as host physiology and genetics, lifestyle, geography may also affect gut microbiota and metabolome composition. It was a preliminary analysis with small sample size and a study of a much larger population with different groups across Asia and the rest of the world would give a better picture of the connection between gut microbiota, metabolites, and diet. However, we confirmed our findings with three separate omics studies (16S rRNA sequencing, fecal and urine metabolomics) which show the reliability of results. Furthermore, the differences in gut microbiota and metabolites based on dietary patterns were in agreement with previous studies. For example, a higher abundance of amino acids, creatinine, and creatine in Chinese consuming meat based diet was in close agreement with studies carried out by Shankar *et al*. (2017) and Stella *et al*.^27,37^. Similarly, in our previous study on gut microbiome by Jain *et al*. (2018) we reported the dominance of *Bacteroidetes and Prevotella* in Indian subjects consuming carbohydrate rich vegetarian diet which was consistent with several previous studies^27,47^. Moreover, various metabolites such as amino acids, phenolics, glycocholic acid were found to be correlated with bacterial genera or species. The microbiome based analysis does not describe the actual microbial activity as it cannot differentiate between alive and dead microbes. This study strengthens our understanding towards links between metabolite signatures with specific bacterial genera or species which shows that fecal and urine metabolome may compliment sequencing-based approaches with a functional readout of the microbiome.

## Materials and Methods

### Chemicals

All chemicals were of analytical grade. Amino acid standard AAS18 (Sigma-Aldrich Chemical Co., St. Louis, MO, USA) was used for the identification of amino acids. Lactic acid, benzoic acid, succinic acid, 4-hydroxybenzaldehyde, 3- phenylpropionic acid, malic Acid, 4-methoxyphenylacetic acid, Gamma-Aminobutyric acid, trans-Cinnamic acid, 3-hydroxybenzoic acid, 4-hydroxybenzoic acid, 4-hydroxyphenylacetic acid, adonitol, hydrocinnamic acid, 3,4-dihydroxyphenylacetic acid, citric acid, D-Fructose, d-Galactose, d-Glucose, 3,4-dihydroxyhydrocinnamic acid, 3-(4-hydroxyphenyl)propionic acid, 1H-indole-3-acetic Acid, D-mannitol, dextrose, sucrose, indole, were purchased from Sigma-Aldrich Chemical Co. (St. Louis, MO, USA) and used for identification purposes. Stock solutions of all the analytical standards were prepared by dissolving the compounds in MilliQ water.

### Recruitment of volunteers

The participants were interviewed and subjects with similar dietary habits were chosen in each group. A total of 16 healthy adults, including 11 Indians and 5 Chinese, were recruited for the current study (Table 3). All the volunteers were university students, ages 22 to 35, studying in Singapore for past 1–3 years. Healthy individuals without any gastrointestinal disorder and who did not use any antibiotics, laxatives or other drugs known to influence gastrointestinal function in the 3 months before the study, were selected^24^. The written informed consent forms and standard questionnaire were taken from the volunteers. They were instructed to maintain their regular diet for a week just before sample collection. Food Frequency Questionnaire (FFQ) was used to recall food diary (Supplementary File 1). Ethical approval was granted by Nanyang Technological University- Institutional Review Board, Singapore. All experiments were performed in accordance with relevant guidelines and regulations.

**Table 3:** Sample information of age, gender, ethnicity, BMI and dietary habits.

Sample ID	Age	Gender	Ethnicity	BMI	Diet
IN1	31	Male	Indian	25.9	**Carbohydrate rich vegetarian:** whole wheat or other whole grains, nuts, rice, lentils, legumes, green vegetables, fruits, dairy products, sweets, ghee, refined flour and fast food
IN2	30	Female	Indian	23	
IN3	30	Male	Indian	26.1	
IN4	30	Female	Indian	22.5	
IN5	23	Female	Indian	23.3	
IN6	26	Female	Indian	23.5	
IN7	27	Male	Indian	25.3	
IN8	26	Female	Indian	22.8	
IN9	30	Male	Indian	24.8	
IN10	27	Female	Indian	21.8	
IN11	23	Female	Indian	22.8	
CH1	26	Male	Chinese	24.6	**Animal fat and protein in addition to carbohydrates:** seafood, fish, chicken, pork, beef, a lot of variety of other meat and animal fat such as lard. rice, noodles, beans, refined grains, white flour, peanut oil
CH2	23	Female	Chinese	21.9	
CH3	22	Female	Chinese	21.8	
CH4	35	Male	Chinese	26.2	
CH5	23	Female	Chinese	21.8	

### Sample collection

All participants were asked to refrain from smoking, eating, drinking for at least 1–2 hour prior to samples collection. Study participants were provided with two different containers: a sterile pot and a 50 mL sterile centrifuge tube. The volunteers were asked to transfer fresh feces from the sterile pot to the tube immediately after defecation and urine samples were collected directly in 50 mL sterile centrifuge tube. The samples were anonymized as, IN1, IN2…IN11 for Indians and CHI, CH2…CH5 for Chinese^24^. Samples were homogenized, 10 g of feces were taken in 50 ml falcon tube and centrifuged (50,000 × g at 10 °C for 2 h), the supernatant is collected^48^. The urine samples in 50 ml falcon tubes were centrifuged to remove any debris (50,000 × g at 10 °C for 15 mins). The fecal water or urine samples were transferred to 1.5 ml Eppendorf tubes and immediately stored at −80 °C freezer prior to metabolite extraction.

### GC-MS sample preparation and metabolite extraction

Fecal water or urine samples were thawed and 100 µl of samples were taken in fresh Eppendorf tubes. Five microliters of 4 mg/mL ribitol dissolved in MilliQ water was added to every sample as an internal standard to correct for any loss of metabolite during the extraction process. A blank with MilliQ water is prepared and treated same as sample. For protein precipitation, 450 µl of acetonitrile/methanol (3:1) was added, vortexed the mixture for 2 minutes and kept at room temperature for 10 minutes. The samples were then centrifuged at 12000 rpm at 4 °C for 20 minutes and the supernatant was transferred to a fresh Eppendorf tube. A second extraction was conducted by adding 200 µl of methanol/water (8:1) to the remaining residue, vortexed for 2 minutes, kept at room temperature for 10 minutes, then centrifuged the mixture at 12000 rpm at 4 °C for 20 minutes. Now the previous supernatant was added to the tube and whole mixture was centrifuged for 5 minutes, transferred the whole supernatant to a fresh Eppendorf tube. The samples were air dried using a heat block at 30 °C for 24 hours. Samples were derivatized by adding 50 µl of 2% methoxyamine HCL in pyridine (ThermoFisher Scientific) and incubated for 1 h at 37 °C. Next, 100 μL of N-methyl-N-(trimethylsilyl)-trifluoroacetamide (MSTFA) with 1% trimethylchlorosilane (TMCS) (Sigma-Aldrich) was added to all samples and incubated at 70 °C for 30 min. Samples were centrifuged for 1 h at room temperature and then transferred to GC-MS glass vials^49^ 0.15 µl of amino acids standard and all other analytical standards (1 mg/ml) were air dried and derivatized same as the samples, and transferred to GC-MS vials.

### GC-MS analysis and metabolites detection

The analysis of samples and standards was done using Agilent Technologies 5973N GC/MS. Metabolites were isolated through a HP-5MS capillary 54 column (30 m × 0.250 mm i.d.; 0.25-μm film thickness; Agilent J&W Scientific). Six times urine and six times fecal sample were run before the actual samples for conditioning of column. Autosampler injected 1 µl of each sample and the separation was performed using the column in splitless mode. The carrier gas was Helium with a flow rate of 1.1 mL/min. Temperatures for inlets and MS source were taken as 250 °C and 230 °C, respectively. The temperature of the oven was kept at 75 °C for 4 min and increased to 280 °C with a rate of 4 °C/min then held for 1.56 min. Mass spectrum was recorded from 40 to 600 m/z with a scan time of 0.2 s.

Data processing and metabolite identification were performed according to the previous study^50^. Briefly, data extraction was performed by GC-MS solution software (GC/MSD Chemstation Data Analysis, Agilent). The total ion chromatogram was obtained, and then mass spectra were identified. The detected metabolites peaks were identified using the NIST 08 mass spectral library (National Institute of Standards and Technology) and the extracted compounds were aligned, normalized according to internal standard ribitol. Peaks with a similarity index more than 80% were used for feature identification. The metabolites of microbial origin were identified using human metabolome database.

### LC-MS metabolomics analysis and metabolites identification

100 µl of fecal water or urine samples were thawed and filtered using 0.22 µm pore size membrane (Jiménez-Girón *et al*. 2015). A blank with methanol is prepared. Five microliters of 4 mg/mL ribitol dissolved in MilliQ water was added to every sample as an internal standard to correct for any loss of metabolite during the extraction process. Metabolomic analysis of filtered solution was performed using Agilent 6550 iFunnel Q-TOF LC/MS system (Agilent Technologies, Santa Clara, CA, USA), operated in both positive and negative ion mode. Six times urine and six times fecal sample were run before the actual samples for conditioning of column.2 µl of samples were injected into an Agilent ZORBAX Rapid Resolution HD SB C18 (2.1 × 100 mm, 1.8 μm) maintained at 45 °C. The flow rate was set at a constant 0.4 ml/min and the pressure was 600 bar. The gradient mobile phase was composed of phase A (water containing 0.1% formic acid) and phase B (acetonitrile containing 0.1% formic acid). The gradient started with 95% A from 0 to 1 min and decreased to 5% from 1 min to 13 min, holding at 5% A till 16 min then turned to 95% in next 10 minutes and holding at 95% A for 4 minutes.

The parameters were the following: capillary voltage 3500 V, nozzle voltage 1000 V, skimmer voltage 65 V, drying gas temperature 200, sheath gas temperature 350, fragmentor voltage 175 V, drying gas flow rate 14 l/min, sheath Gas flow rate 11 l/min, nebulizer pressure 35 psi. MS data were recorded across the range of 50− 1700 m/z at 1.5 spectra/s. Each sample was injected and analysed two times.

All raw data extracted and processed using Agilent MassHunter Qualitative Analysis B.07.00 software. A list of peak areas, retention time and mass to charge (m/z) were obtained and metabolites were identified by comparing the data to selected databases, namely, KEGG, HMDB, and METLIN.

### Statistical analysis

Multivariate statistical analysis was performed using MetaboAnalyst 3.0^51^. Partial least squares discriminant analysis was performed to see the difference between metabolite profiles of Indian and Chinese adults. The VIP >1 was taken to identify the features significantly differentiating between Indian and Chinese adults, then fold change ratio was obtained for each feature. Hierarchical cluster analysis heat map was obtained using ward clustering algorithm and Euclidean distance calculation to further confirm the results of PLS-DA and to show the distribution of metabolites among all individuals. Pathways analysis was performed and correlations between microbiome and metabolites were obtained using Pearson correlation coefficient. The microbiome data was obtained from our previous study^24^.

### Ethical approval and Informed consent

Ethical approval was granted by Nanyang Technological University—Institutional Review Board, Singapore. The written informed consent forms were taken from the volunteers.

## Supplementary information

Figure S1

Table S1

Table S2

Table S3

Table S4

Supplementary file 1

## Data Availability

The metabolomics datasets generated during the current study are available from the corresponding author on reasonable request. The 16S rRNA gene sequencing data used in the paper have been deposited in the National Omics Data Encyclopaedia (http://www.biosino.org/node/index) under Accession Number NODEP00371763.
