# Supplementary material for: An untargeted fecal and urine metabolomics analysis of the interplay between the gut microbiome, diet and human metabolism in Indian and Chinese adults
Source: Sci Rep. 2019 Jun 24;9:9191. doi: 10.1038/s41598-019-45640-y (PMC6591403; doi:10.1038/s41598-019-45640-y)
Supplement: Supplementary file 1 — Figure S1 [file 41598_2019_45640_MOESM1_ESM.docx]

**Journal Name: Scientific Reports**

**Manuscript Title: An untargeted fecal and urine metabolomics analysis of the interplay between the gut microbiome, diet and human metabolism in Indian and Chinese adults**

**Abhishek Jain^a,b^,  Xin Hui Li ^d^, Wei Ning Chen^c,*^**

^a^ Interdisciplinary Graduate School, Nanyang Technological University, 50 Nanyang Avenue, Singapore 639798, Singapore

^b^ Advanced Environmental Biotechnology Centre, Nanyang Environment & Water Research Institute, Nanyang Technological University, 1 CleanTech Loop, Singapore 637141, Singapore

^c^ School of Chemical and Biomedical Engineering, Nanyang Technological University, 62 Nanyang Drive, Singapore 637459, Singapore

^d^ Zhong Feng International, Hengyang City, China

Author

Abhishek Jain

E-mail address: [abhishek017@e.ntu.edu.sg](mailto:abhishek017@e.ntu.edu.sg)

Xin Hui Li

E-mail address: [1522092868@qq.com](mailto:1522092868@qq.com)

^*^Corresponding author

Wei Ning Chen

School of Chemical and Biomedical Engineering, Nanyang Technological University

62 Nanyang Drive, N1.2-B1-07, Singapore 637459

E-mail address: [wnchen@ntu.edu.sg](mailto:wnchen@ntu.edu.sg)

**Fig. S1** A representative GC-MS chromatogram of fecal extract
