# Supplementary material for: An untargeted fecal and urine metabolomics analysis of the interplay between the gut microbiome, diet and human metabolism in Indian and Chinese adults
Source: Sci Rep. 2019 Jun 24;9:9191. doi: 10.1038/s41598-019-45640-y (PMC6591403; doi:10.1038/s41598-019-45640-y)
Supplement: Supplementary file 6 — Supplementary file 1 [file 41598_2019_45640_MOESM6_ESM.docx]

**Journal Name: Scientific Reports**

**Manuscript Title: An untargeted fecal and urine metabolomics analysis of the interplay between the gut microbiome, diet and human metabolism in Indian and Chinese adults**

**Abhishek Jain^a,b^,  Xin Hui Li ^d^, Wei Ning Chen^c,*^**

^a^ Interdisciplinary Graduate School, Nanyang Technological University, 50 Nanyang Avenue, Singapore 639798, Singapore

^b^ Advanced Environmental Biotechnology Centre, Nanyang Environment & Water Research Institute, Nanyang Technological University, 1 CleanTech Loop, Singapore 637141, Singapore

^c^ School of Chemical and Biomedical Engineering, Nanyang Technological University, 62 Nanyang Drive, Singapore 637459, Singapore

^d^ Zhong Feng International, Hengyang City, China

Author

Abhishek Jain

E-mail address: [abhishek017@e.ntu.edu.sg](mailto:abhishek017@e.ntu.edu.sg)

Xin Hui Li

E-mail address: [1522092868@qq.com](mailto:1522092868@qq.com)

^*^Corresponding author

Wei Ning Chen

School of Chemical and Biomedical Engineering, Nanyang Technological University

62 Nanyang Drive, N1.2-B1-07, Singapore 637459

E-mail address: [wnchen@ntu.edu.sg](mailto:wnchen@ntu.edu.sg)

Food Frequency Questionnaire (FFQ)

Subject no:

Name:

Age:

Marital Status:

Gender: M / F

Race: Chinese / Indian

Contact no/ email address:

**Breads and Cereals**

| Food Item | | Portion | Number of times eaten | | | |
| --- | --- | --- | --- | --- | --- | --- |
| How often do you eat the following: | |  | Per day | Per week | Per month | Rarely/Never |
| **Bread** | | | | | | |
| 1 | White bread | 1 slice or 1 piece |  |  |  |  |
| 2 | Whole meal bread | 1 slice or 1 piece |  |  |  |  |
| 3 | Bread with fruits and nuts | 1 slice or 1 piece |  |  |  |  |
| **Bread spreads used** | | | | | | |
| 4 | Butter | 1 tsp (D2) |  |  |  |  |
| 5 | Margarine | 1 tsp (D2) |  |  |  |  |
| 6 | Peanut butter | 1 tsp (D2) |  |  |  |  |
| 7 | Jams/honey | 1 tsp (D2) |  |  |  |  |
| 8 | Kaya | 1 tsp (D2) |  |  |  |  |
| 9 | Lard | 1 tsp (D2) |  |  |  |  |
| **Other types of breads** | | | | | | |
| 10 | Roti/Chapati (wheat)  Naan  Prata (All-purpose flour/Maida) | 1 piece |  |  |  |  |
| 11 | Thosai  Idli  Vadai  Others | 1 piece |  |  |  |  |
| 12 | French toast/roti telur/roti john | 1 piece |  |  |  |  |
| 13 | Bread buns with coconut/curry/meat fillings | 1 piece |  |  |  |  |
|  | **Cereals** | | | | | |
| 14 | Plain/flavoured breakfast cereals | 4 dsp (D1) |  |  |  |  |
| 15 | Mixed (with fruit/nuts) breakfast cereals | 4 dsp (D1) |  |  |  |  |
| **For participants who consume breakfast cereals (#14, 15):** | | | | | | |
| A | You have indicated that you eat breakfast cereals. How often do you have breakfast cereals made from whole-grains? | 4 dsp (D1) |  |  |  |  |
| B | Oats/oatmeal (raw) | 4 dsp (D1) |  |  |  |  |

**Rice and Porridge**

| Food Item | | Portion | Number of times eaten | | | |
| --- | --- | --- | --- | --- | --- | --- |
| How often do you eat the following: | |  | Per day | Per week | Per month | Rarely/Never |
| **Bread** | | | | | | |
| 16 | white rice/red rice/brown rice | 1B1 |  |  |  |  |
| 17 | Plain porridge | 1B1 |  |  |  |  |
| **Flavoured rice/porridge** | | | | | | |
| 18 | Fried rice | 1B1 |  |  |  |  |
| 19 | Chicken/duck rice/Chicken biryani | 1 Portion |  |  |  |  |
| 20 | Mui fan/curd rice | 1 Portion |  |  |  |  |
| 21 | Nasi briyani | 1 Portion |  |  |  |  |
| 22 | Nasi lemak | 1 Portion |  |  |  |  |
| 23 | Claypot rice | 1 Portion |  |  |  |  |
| 24 | Glutinuous rice | 1 Portion |  |  |  |  |
| 25 | Flavored porridge  (e.g. chicken, pork, duck, fish) | 1 Portion |  |  |  |  |

**Noodles (rice noodles, wheat noodles, bean noodles, pasta)**

| Food Item | | Portion | Number of times eaten | | | |
| --- | --- | --- | --- | --- | --- | --- |
| How often do you eat the following: | |  | Per day | Per week | Per month | Rarely/Never |
| **Soup noodles** | | | | | | |
| 26 | Fishball/yong tau foo/wanton/prawn/ beef/chicken/ fish slice | 1 Portion |  |  |  |  |
| **For participants who consume Soup noodles(#26):** | | | | | | |
| C | You have indicated that you eat noodles in soup. How often do you have soup noodles prepared using brown rice beehoon? | 1portion |  |  |  |  |
| **N oodles in gravy** | | | | | | |
| **27** | Lor mee/mee rebus | 1 Portion |  |  |  |  |
| 28 | Laksa lemak | 1 Portion |  |  |  |  |
| 29 | Mee siam (with coconut milk) | 1 Portion |  |  |  |  |
| **Other Noodles** | | | | | | |
| 30 | Instant noodles | 1 Portion |  |  |  |  |
| 31 | Boiled noodles / spaghetti / pasta (plain) | 1 Portion |  |  |  |  |
| 32 | Boiled noodles / spaghetti / pasta with tomato sauce | 1 Portion |  |  |  |  |
| 33 | Boiled noodles / spaghetti / pasta with cream white sauce | 1 Portion |  |  |  |  |
| **For participants who consume boiled spaghetti/pasta (#30-33):** | | | | | | |
| D | You have indicated that you eat boiled spaghetti/pasta. How often do you have spaghetti/pasta prepared using wholemeal spaghetti/pasta? | 1 portion |  |  |  |  |

**Vegetables and Beancurd**

| Food Item | | Portion | Number of times eaten | | | |
| --- | --- | --- | --- | --- | --- | --- |
| How often do you eat the following: | |  | Per day | Per week | Per month | Rarely/Never |
| **Pale green leafy vegetables (cabbage, pak choy, lettuce, beansprouts, cauliflower etc)** | | | | | | |
| 34 | Stir fried, plain | ½ cup |  |  |  |  |
| 35 | Stir fried, with meat/ seafood | ½ cup |  |  |  |  |
| 36 | Stir fried in oyster sauce | ½ cup |  |  |  |  |
| 37 | Curry/lemak | ½ cup |  |  |  |  |
| 38 | Raw/steamed/in soup | 1 cup |  |  |  |  |
| **Dark green leafy vegetables (spinach, kai lan, chye sim, kangkong, broccoli etc)** | | | | | | |
| 39 | Stir fried, plain | ½ cup |  |  |  |  |
| 40 | Stir fried, with meat/ seafood | ½ cup |  |  |  |  |
| 41 | Stir fried in oyster sauce | ½ cup |  |  |  |  |
| 42 | Stir fried in sambal belacan/dried prawns | ½ cup |  |  |  |  |
| 43 | Raw/steamed/in soup | 1 cup |  |  |  |  |
| **Tomatoes, carrots, red/yellow peppers** | | | | | | |
| 44 | Stir fried, plain | ½ cup |  |  |  |  |
| 45 | Stir fried, with meat/ seafood | ½ cup |  |  |  |  |
| 46 | Curry/lemak | ½ cup |  |  |  |  |
| 47 | Raw/steamed/in soup | 1 cup |  |  |  |  |
| **Legumes/pulses, e.g. beans, peas** | | | | | | |
| 48 | Stir fried, plain | ½ cup |  |  |  |  |
| 49 | Stir fried in oyster sauce | ½ cup |  |  |  |  |
| 50 | Stir fried in sambal belacan | ½ cup |  |  |  |  |
| 51 | Dried legumes (e.g.dhal, dried beans) in gravy | ½ cup |  |  |  |  |
| 52 | Raw/steamed/boiled | ½ cup |  |  |  |  |
| **Mixed vegetables** | | | | | | |
| **53** | Stir fried, plain | ½ cup |  |  |  |  |
| 54 | Stir fried, with meat/ seafood | ½ cup |  |  |  |  |
| 55 | Stir fried in oyster sauce | ½ cup |  |  |  |  |
| 56 | Curry/lemak | ½ cup |  |  |  |  |
| 57 | Raw/steamed/in soup/ Chinese rojak | 1 cup or  1 serving |  |  |  |  |
| **Tofu/beancurd** | | | | | | |
| 58 | Fried | ½ square |  |  |  |  |
| 59 | Steamed/ in soups | ½ square |  |  |  |  |
| **Roots/stems (potatoes, sweet potatoes, corn etc)** | | | | | | |
| 60 | Stir fried | 1 cup |  |  |  |  |
| 61 | Steamed | 1 cup |  |  |  |  |

**Fruits**

| Food Item | | Portion | Number of times eaten | | | |
| --- | --- | --- | --- | --- | --- | --- |
| How often do you eat the following: | |  | Per day | Per week | Per month | Rarely/Never |
| 61 | Orange/red/yellow fresh fruits | 1 serving |  |  |  |  |
| 62 | Other fresh fruits | 1 serving |  |  |  |  |
| 63 | Fresh fruit juice | 1 cup |  |  |  |  |
| 64 | Bananas | 1 medium |  |  |  |  |
| 65 | Durians | 5 seeds |  |  |  |  |
| 66 | Canned fruits | ½ cup |  |  |  |  |
| 67 | Mixed fruits (dried) | 1 serving |  |  |  |  |

**Poultry**

| Food Item | | Portion | Number of times eaten | | | |
| --- | --- | --- | --- | --- | --- | --- |
| How often do you eat the following: | |  | Per day | Per week | Per month | Rarely/Never |
| 68 | Steamed | 1 serving |  |  |  |  |
| 69 | Fried | 1 serving |  |  |  |  |

**Meat**

| Food Item | | Portion | Number of times eaten | | | |
| --- | --- | --- | --- | --- | --- | --- |
| How often do you eat the following: | |  | Per day | Per week | Per month | Rarely/Never |
| **Meat-lean** | | | | | | |
| 70 | Steamed | 1 serving |  |  |  |  |
| 71 | Fried | 1 serving |  |  |  |  |
| **Meat-lean and fat** | | | | | | |
| 72 | Steamed | 1 serving |  |  |  |  |
| 73 | Fried | 1 serving |  |  |  |  |
| **Meat – preserved/cured** | | | | | | |
| 74 | Sausages | One |  |  |  |  |
| 75 | Ham | 1 slice |  |  |  |  |
| 76 | Bacon | 1 slice |  |  |  |  |
| 77 | Canned (e.g. luncheon meat, corned beef) | Size of 4 square of chocolate |  |  |  |  |
| 78 | Liver and other innards | Size of 4 square of chocolate |  |  |  |  |

**Fish/Seafood**

| Food Item | | Portion | Number of times eaten | | | |
| --- | --- | --- | --- | --- | --- | --- |
| How often do you eat the following: | |  | Per day | Per week | Per month | Rarely/Never |
| **Fish** | | | | | | |
| 79 | Steamed | 1 serving |  |  |  |  |
| 80 | Fried | 1 serving |  |  |  |  |
| 81 | Raw | 1 serving |  |  |  |  |
|  | **Other sea-food** | | | | | |
| 82 | Steamed | 1 serving |  |  |  |  |
| 83 | Fried | 1 serving |  |  |  |  |
| 84 | Raw | 1 serving |  |  |  |  |

**Eggs**

| Food Item | | Portion | Number of times eaten | | | |
| --- | --- | --- | --- | --- | --- | --- |
| How often do you eat the following: | |  | Per day | Per week | Per month | Rarely/Never |
| **Whole eggs (including salted and century eggs)** | | | | | | |
| 85 | Boiled/poached/in soup/steamed | 1 egg |  |  |  |  |
| 86 | Fried/scrambled | 1 egg |  |  |  |  |
| **Egg whites, only** | | | | | | |
| 87 | Boiled/poached/in soup/steamed | 1 egg |  |  |  |  |
| 88 | Fried/scrambled | 1 egg |  |  |  |  |

**Desserts/Local Snacks**

| Food Item | | Portion | Number of times eaten | | | |
| --- | --- | --- | --- | --- | --- | --- |
| How often do you eat the following: | |  | Per day | Per week | Per month | Rarely/Never |
| 89 | Fried snacks (e.g. you tiao, goreng pisang, Indian rojak, Samosa, Pakodas) | 1 piece |  |  |  |  |
| 90 | Dim sum – steamed (e.g chee cheong fun, dumplings, rice dumplings) | 1 serving |  |  |  |  |
| 91 | Dim sum – fried/deep fried (e.g. fried carrot cake, wanton, char siew puff) | 1 piece |  |  |  |  |
| 92 | Sweet Indian snacks (e.g. burfi, halwa) | 1 piece |  |  |  |  |

**Biscuits, Pastries and Cakes**

| Food Item | | Portion | Number of times eaten | | | |
| --- | --- | --- | --- | --- | --- | --- |
| How often do you eat the following: | |  | Per day | Per week | Per month | Rarely/Never |
| 93 | Plain biscuits | 2 pieces |  |  |  |  |
| 94 | Cream filled biscuits/shortbread | 2 pieces |  |  |  |  |
| 95 | Puff/flaky pastries (croissants, baked curry puffs etc) | 1 piece |  |  |  |  |
| 96 | Plain butter cake/fruit cake | 1 piece |  |  |  |  |
| 97 | Sponge cakes | 1 piece |  |  |  |  |
| 98 | Cream cakes | 1 piece |  |  |  |  |

**Fast Foods**

| Food Item | | Portion | Number of times eaten | | | |
| --- | --- | --- | --- | --- | --- | --- |
| How often do you eat the following: | |  | Per day | Per week | Per month | Rarely/Never |
| 99 | Burgers, with beef or chicken | 1 serving |  |  |  |  |
| 100 | Burgers, fish | 1 serving |  |  |  |  |
| 101 | French fries | 1 small serving |  |  |  |  |
| 102 | Pizza | 2 slices |  |  |  |  |
| 103 | Mashed potato with gravy | 1 regular |  |  |  |  |

**Sweetened beverages**

| Food Item | | Portion | Number of times eaten | | | |
| --- | --- | --- | --- | --- | --- | --- |
| How often do you eat the following: | |  | Per day | Per week | Per month | Rarely/Never |
| 104 | Sweetened beverages (e.g. soft drinks, packet drinks, yoghurt drinks) | 1 G2 |  |  |  |  |

**All types of Nuts**

| Food Item | | Portion | Number of times eaten | | | |
| --- | --- | --- | --- | --- | --- | --- |
| How often do you eat the following: | |  | Per day | Per week | Per month | Rarely/Never |
| 105 | Raw | ½ M1 or 1 small pkt |  |  |  |  |
| 106 | Dry roasted | ½ M1 or 1 small pkt |  |  |  |  |
| 107 | Fried | ½ M1 or 1 small pkt |  |  |  |  |

**Titbits/Snacks**

| Food Item | | Portion | Number of times eaten | | | |
| --- | --- | --- | --- | --- | --- | --- |
| How often do you eat the following: | |  | Per day | Per week | Per month | Rarely/Never |
| 108 | Fried salty snacks (crisps, prawn crackers, keropok, salted biscuits etc) | 1 small packet |  |  |  |  |
| 109 | Ice cream | 1 scoop |  |  |  |  |
| 110 | Chocolate | 4 squares |  |  |  |  |

## Milk & Dairy Products

| Food Item | | Portion | Number of times eaten | | | |
| --- | --- | --- | --- | --- | --- | --- |
| How often do you eat the following: | |  | Per day | Per week | Per month | Rarely/Never |
| 111 | Coffee(with & without milk) | 1 M1 –D/2 tsp |  |  |  |  |
| 112 | Tea(with & without milk) | 1 M1 –D/2 tsp |  |  |  |  |
| 113 | Malt beverages (e.g. hot chocolate, Horlicks®, Milo®, Ovaltine®) | 1 M1 –D/2 tsp |  |  |  |  |
| 114 | Full cream milk* (fresh, UHT, powder) | 1 G2* |  |  |  |  |
| 115 | Low fat milk* (fresh, UHT, powder) | 1 G2* |  |  |  |  |
| 116 | Skimmed milk* (fresh, UHT, powder) | 1 G2* |  |  |  |  |
| 117 | Regular yoghurt | 1 G1 |  |  |  |  |
| 118 | Low fat (including frozen yoghurt) | 1 G1 |  |  |  |  |
| 119 | Cheese | 1 slice/4dsp |  |  |  |  |
| 120 | Paneer | 4 cubes |  |  |  |  |

**Soya Products**

| Food Item | | Portion | Number of times eaten | | | |
| --- | --- | --- | --- | --- | --- | --- |
| How often do you eat the following: | |  | Per day | Per week | Per month | Rarely/Never |
| 121 | Soya milk (fresh/packet/can) | 1 G2 |  |  |  |  |
| 122 | Soya beancurd (tau huay) | 1 B1 |  |  |  |  |

**Legend**

B1: 1 rice bowl (volume of 300ml)

B2: 1 soup bowl (volume of 700ml)

D1: 1 dessert spoon (10ml)

D2: 1 teaspoon (5ml)

G1: Glass (200ml)

G2: Glass (300ml)

M1: Mug (200ml)
